# Eliminating Iodine Deficiency in China: Achievements, Challenges and Global Implications

**DOI:** 10.3390/nu9040361

**Published:** 2017-04-05

**Authors:** Dianjun Sun, Karen Codling, Suying Chang, Shubin Zhang, Hongmei Shen, Xiaohui Su, Zupei Chen, Robert W. Scherpbier, Jun Yan

**Affiliations:** 1 Centre for Endemic Disease Control, China Chinese Centre for Disease Control and Prevention, Harbin Medical University, 157 Baojian Road, Harbin 150081, China; hrbmusdj@163.com (D.S.); shenhm119@126.com (H.S.); Suxiaohui8@163.com (X.S.); 2 United Nations Children’s Fund, 12 Sanlitun Lu, Beijing 100600, China; karen@publicnutritionsolutions.com or kcodling@ign.org (K.C.); schang@unicef.org (S.C.); 3 Iodine Global Network, P.O. Box 51030, 375 des Epinettes, Ottawa, ON K1E 3E6, Canada; zpchentj125@sina.com (Z.C.); rscherpbier@unicef.org (R.W.S.); 4 Parasitic and Endemic Disease Prevention and Control Division of the National Health and Family Planning Commission of China, 14 Zhichun Road, Beijing 100088, China; zsbin@126.com

**Keywords:** China, iodine deficiency, salt iodization, iodine status

## Abstract

The prevention of iodine deficiency through salt iodization has been recognized as a global success story, and China stands at the forefront of this achievement with one of the most successful programs in the world. High level political commitment, national mandatory legislation, a state-managed edible salt industry and a complex and highly sophisticated surveillance system have facilitated the success of the program. Challenges have arisen however, including: (i) concern that adequate iodine status in pregnant women cannot be achieved without causing above adequate iodine intakes in children; (ii) declining iodine intake as a result of reductions in salt consumption and increased consumption of processed foods, which may not be made with iodized salt; (iii) the existence of areas with high iodine content in the water; and (iv) declines in household use of iodized salt due to concerns about excess iodine intake and thyroid disease. This article reviews the achievements and challenges of the Chinese Iodine Deficiency Disorders (IDD) Elimination Program and reflects on lessons learned and implications for other national salt iodization programs.

## 1. Introduction

Iodine is required for the production of thyroid hormone. However, deficiencies occur in significant areas of the world due to the erosion of iodine from the soil by loss of vegetation, overgrazing and deforestation [1]. Deficiencies in intake cause a spectrum of growth, developmental and functional morbidities across the life course, termed iodine deficiency disorders (IDD) [2]. In all age groups, iodine deficiency can cause goiter, an enlargement of the thyroid gland, as it adapts to chronic iodine deficiency [3]. Iodine deficiency in childhood causes intellectual impairment and growth retardation and is an important cause of preventable mental impairment worldwide [4]. Chinese researchers have found that children living in iodine-deficient areas with no iodine supplementation/fortification had an average of 12.45 intelligence quotient points less than children living in an iodine-sufficient environment [5]. IDD in pregnant women causes increased pregnancy loss and infant mortality, cretinism and neonatal hypothyroidism in their children [3]. Even mild deficiency in women during pregnancy has been found to cause subsequent educational and cognitive impairments in their children [6,7]. Iodine deficiency is one of the most common micronutrient deficiencies in the world. In the mid-1990s, the World Health Organization (WHO) estimated that there were in excess of 2.2 billion people from 130 countries at risk of IDD [8]. Conversely however, excess iodine can also cause iodine disorders, in particular goiter, thyroiditis, hyper- and hypo-thyroidism and thyroid cancer [9].

Universal salt iodization (USI) is the recommended strategy for controlling iodine deficiency [4] and requires that all food-grade salt, including food industry and household salt, be iodized [10] in order to address the lack of iodine in the environment. The United Nations Children’s Fund (UNICEF) estimates that over 140 countries are implementing salt iodization programs [11], and a database maintained by the Iodine Global Network (IGN) indicates that 129 out of 197 countries have mandatory legislation for the iodization of at least household/table salt or salt for food processing [12]. Today, 75% of households worldwide use iodized salt [13], and as a result, the number of iodine-deficient countries has decreased from 110 in 1993 to 15 in 2016 [14].

Salt iodization is recommended by organizations such as the WHO, UNICEF and IGN, (previously called the International Council for the Control of IDD (ICCIDD)), because: (i) salt is widely consumed by virtually all population groups in all countries, with little seasonal variation in consumption; (ii) salt production is generally limited to a few centers, facilitating quality control; (iii) technology for salt iodization is well established and relatively easy to transfer to less developed countries; (iv) iodization does not affect the organoleptic properties of salt, and therefore, consumer acceptability is high; and (v) iodization is very inexpensive [15]. A review, commissioned by WHO, on the effects and safety of salt iodization concluded that iodized salt has a large effect on reducing the risk of goiter, cretinism, low cognitive function and iodine deficiency [15]. Salt iodization is also one of the most cost-effective nutrition interventions; having an estimated benefit: cost ratio of 30:1 from improved economic productivity due to averting cognitive deficiencies in children born to mothers who would otherwise have goiter or subclinical iodine deficiency. This rate does not include additional benefits due to averting stillbirths, etc. [16]. 

## 2. The Chinese Iodine Deficiency Disorder Elimination Programme

Iodine deficiency has been recognized as a severe public health problem in China since the 1930s. Data collected between the 1960s and 1990s recorded iodine deficiency of varying degrees in all of China’s provinces [17,18]. In many endemic areas, 5%–15% of children suffered from mild retardation (intelligence quotient of 50–69) [19].

As a result of these data demonstrating a significant IDD problem, salt iodization interventions were started in endemic areas of China in the 1960s. In 1993, as a follow up to the 1990 United Nations Summit for Children, the National Advocacy Programme for the Elimination of IDD by 2000 was launched. It was largely the result of a decision by Vice Premier Zhu Rongji, who, as an economist, was concerned about the implications of IDD on national human and economic development [20]. Numerous other countries also initiated salt iodization and passed mandatory salt iodization legislation around this time [21].

While most national IDD programs have simply adopted the WHO targets of IDD elimination, as indicated by optimal median urinary iodine concentration (MUIC), a measure of iodine intake, in school-age children and >90% of household coverage with adequately iodized salt [1], the Chinese National IDD Elimination Programme has more sophisticated targets and has been implemented in four phases, as shown in Table 1 below.

The National IDD Elimination Programme is officially coordinated by the Vice Minister, but on a day-to-day basis, the program is managed by the National Health and Family Planning Commission (NHFPC), the equivalent of the Ministry of Health in China. Mandatory universal salt iodization is the main strategy adopted for the elimination of IDD in China. Complementary interventions include routine and emergency iodized oil capsule distribution for high-risk populations (pregnant and lactating women) and free distribution of iodized salt in some of the low coverage provinces. Mandatory salt iodization started in 1994 when the “Regulation on Salt Iodization to Eliminate IDD” [24] was issued by the State Council. The Regulation requires all edible salt to be iodized, including salt for food processing and animal consumption. 

Implementation of the salt iodization legislation has been facilitated by the fact that China is self-sufficient in both industrial and edible salt, and an official monopoly exists for edible salt in China. Moreover, under the Regulation on Management of the Edible Salt Industry [25] of 1990, all aspects of salt production, distribution and sale are controlled and managed by the state. Only authorized entities are allowed to produce, distribute or market edible salt, and production quotas and prices are controlled. The monopoly is managed by the Ministry of Industry and Information Technology and China National Salt Industry Corporation (CNSIC), a state-owned enterprise. A specific objective of the edible salt monopoly is to facilitate salt iodization, and the sale of salt that does not meet national standards is prohibited. No other country has a similarly managed edible salt industry. Additionally, considerable funding was provided to the salt industry by the government at the start of the program for equipment for iodization and packaging [26], and iodine fortification, potassium iodate, is provided free by the Ministry of Finance.

The national standard for edible salt specifies the required iodization level. The iodization level has been changed three times in order to fine-tune the program. When the salt iodization program was first established in 1994, the average iodization level at production was set at 50 mg/kg, with a minimum of 40 mg/kg for salt leaving the factory. At retail, the minimum level was 30 mg/kg, and at the household, it was 20 parts per million (ppm). In 1996, a maximum iodization level of 60 mg/kg was established, as salt in some regions was found to have excessive levels of iodine [27,28]. In 2000, the national standard was changed to be 35 ± 15 ppm (20–50 ppm) at the production level, as the median urinary iodine level of children was felt to be too high [29]. The most recent change in the standard took place in 2011 [30]. The iodization level was further reduced to 20–30 ppm ± 30% (14–39 ppm), and provinces were authorized to adopt one of three standards within this national range. Fourteen of China’s 31 provinces selected the middle range of 18–33 ppm (25% ± 30%); 12 selected the highest range of 21–39 ppm (30% ± 30%); and none selected the lowest range of 14–26 ppm (20% ± 30%). Five provinces selected a combination of standards, either different standards in different prefectures (lower administrative structure than provinces) or a higher standard for pregnant women and a lower one for the general population (salt with the higher amount of iodine is labelled as being for pregnant women). This latest change was again made because the urinary iodine level of school-age children was felt to be too high, being above 200 μg/L in 21 provinces, including four in which it was above 300 μg/L in 2011 [31]. Provincial standards were permitted in recognition of the size of China and variations in iodine status between provinces. Here, again, China differs from other USI programs, which only have one national standard that applies to all household salt. Within Asia, the salt iodization standard has only been amended once in the Philippines in 2013 (higher) and once in Sri Lanka in 2005 (lower). Most other countries have maintained the same standard as when legislation was first issued [21].

A complex and sophisticated regulatory monitoring and surveillance system supports the Chinese National IDD Elimination Program. Routine household surveys monitor the quality of salt, household consumption of adequately iodized salt and the iodine status of the population. In addition, the government has undertaken complementary evaluations at key stages of the program and special “investigations” of specific issues to provide supplementary information. 

The quality of salt is ensured through a variety of regulations and requirements. Authorized salt producers are required to meet “quality management technical specifications” and to implement stringent internal quality assurance procedures to ensure that all salt is adequately iodized. Wholesalers must also be licensed and undertake quality testing. External monitoring systems control the availability of illegal salt. External regulatory monitoring and enforcement by government is probably the component of the program that receives the least attention in China, as it is not needed in the unique context of the government-regulated Chinese edible salt industry; there is no competition between enterprises, and the industry regulates itself and consistently produces a high-quality product. Additionally, all are large, sophisticated entities with high capacity to iodize and access to government-supplied potassium iodate. On the contrary, in other countries, such as several neighboring countries, significant proportions of salt are non-iodized or inadequately iodized because of low capacity for iodization and internal quality control and/or conscious efforts to avoid iodization to save costs on potassium iodate, which salt producers must pay for. The situation is exacerbated by weak external regulatory monitoring and enforcement by authorities because of large numbers of salt processors, insufficient resources and generally weak food control systems [32]. 

Every year in China, “national salt monitoring” is undertaken by county authorities to assess household use of iodized salt. Salt samples, collected from a representative sample of households in the county, are analyzed by titration for iodine content. This provides an annual assessment of household use of adequately iodized salt at the county, province and national level. In addition, “intensified monitoring” was undertaken until 2012 in provinces that had not yet achieved >90% coverage with adequately iodized salt and in target areas of other provinces where the national salt monitoring revealed problems. These were areas near raw salt production, areas unable to undertake salt monitoring and remote and poor areas with low iodized salt coverage.

Data on the iodine status of the population is assessed through the National IDD Survey. The survey has been implemented in 1995, 1997, 1999, 2002, 2005 and 2011. A survey was also undertaken in 2014, but the results of this survey have not yet been published. The National IDD Survey assesses goiter and MUIC in school-age children and, starting from 2011, MUIC in pregnant and lactating women. It also collects data on household use of iodized salt, with iodine content assessed by titration. The data collected are representative of the provincial level. 

A third component of the surveillance system is “High Risk Area Monitoring”, which was initiated in 2008. The objective of this component of the system is to monitor IDD epidemic situations and identify the re-emergency of IDD. High-risk areas are those with iodized salt coverage <80% and a history of endemic cretinism. High Risk Area Monitoring is undertaken at the county level and includes a search for people with cretinism, assessment of urinary iodine in reproductive-age women and goiter rate in school-age children. Iodized oil capsules are distributed to reproductive-age women and children in counties considered at risk on the basis of this monitoring. 

In addition, several special studies have been undertaken to provide supplementary information to the surveillance system, and evaluations were undertaken in 2000, 2007 and 2010 to assess achievement of the goals of the various phases of the program. 

In 2005, following the identification of areas with high levels of iodine in a number of provinces in the former flood plain of the Yellow River, a comprehensive survey was undertaken in 11 provinces. The survey identified 735 townships in 109 counties in 9 provinces as having water iodine levels >150 μg/L [33]. Some 31 million people live in these areas; 2% of the total population. These townships were designated as high water iodine (HWI), areas and an information notice from the Ministry of Health (now the NHFPC) and the Ministry of Industry and Information Technology in 2009 directed that such areas should receive non-iodized salt [34]. In 2007, the National Salt Monitoring component of the surveillance system started monitoring the coverage of non-iodized salt in these areas, and in 2012, the MUIC and goiter rate of school-age children was added. Prior to 2007, HWI areas were excluded from the monitoring system and were treated as ”blind counties”. The situation in HWI areas was further investigated in 2011/2012. The investigation recommended that the demarcation of HWI areas should be reduced from 150 μg/L to 100 μg/L because at these water iodine levels, children and pregnant women were found to have urinary iodine levels indicating excess iodine intake. The investigation also concluded that the supply of non-iodized salt was not sufficient to ensure optimum iodine nutrition; water improvement measures were also necessary [35].

A special survey was undertaken in 101 high-risk counties for iodine deficiency in 2007. It found that 26 remained at high risk due to easy access to raw salt [36]. Iodized oil capsules were provided, and strategies were developed to increase access to adequately iodized salt. 

A survey of iodine intake was undertaken in four coastal provinces in 2009. The survey was in response to concerns that people in coastal provinces might be consuming too much iodine through a combination of seafood and iodized salt. Data from this survey were analyzed together with data from a nationally-representative total diet survey in 2007 and another one in Beijing in 2009. The analysis indicated that iodine intake in coastal provinces was lower than nationally, and aquatic foods contributed only 11.6% of the daily total of iodine intake, while iodized salt contributed about two-thirds of iodine intake [37]. Moreover, the analysis calculated that in the four coastal provinces studied in 2009, while 19.2% and 18.2% of children 2–7 and 8–12 years old consumed iodine above the upper limit, 31.5% of reproductive-age women consumed less than the WHO-recommended daily allowance for iodine.

The data collected by the surveillance system and the additional special studies, investigations and evaluations have provided data on the current status of the program and enabled evaluation against the program targets. This has also provided critical information for fine-tuning and adaptation of the program and has been used to explore questions and concerns about the program. 

The surveillance and evaluation system for the Chinese IDD Elimination Programme is very elaborate compared to that in most other countries. Comprised, as noted above, of annual county-level monitoring, periodic national surveys with provincial representation, additional complementary investigations and evaluations at each phase of the program, the system provides a wealth of information that has been used to adjust, strengthen and perfect the program. In contrast, in most other countries, surveillance and evaluation systems of salt iodization/IDD programs are limited to periodic national IDD surveys or the inclusion of selected IDD/USI indicators into national surveys, such as the Demographic Health Surveys or Multiple Indicator Cluster Surveys. From such surveys, countries are usually able to obtain periodic assessments of household coverage of iodized salt. Better designed and resourced surveys assess iodine content quantitatively (e.g., by titration), rather than qualitatively, and if the sample size is adequate, can obtain sub-national representation. Urinary iodine concentration is even less frequently monitored, and when it is, representation may be only at the national level and only in one population group, often school-age children. For example, India, despite implementing mandatory salt iodization since 1998, only measured urinary iodine in a national survey for the first time in 2015 [38]. 

The Chinese IDD Elimination Programme has also been well resourced and supported. While the program is almost 100% government-funded currently, in the initial stages, considerable external support was provided. The Australian government provided technical and financial support under a Memorandum of Understanding that started in 1986. This support helped to establish the monitoring and surveillance system, trained many staff, supported research and facilitated the scale up of pilot interventions to the national program [17]. The initial phase of the national program, from 1995, was budgeted at US$152 million [39], with significant allocations for building the iodization capacity of the salt industry. While the World Bank provided US$27 million in loan and credit, the Government of China, including the State Development Bank, Salt Industry Development Bank and provincial governments, was accountable for the remainder [40]. UNICEF, the United Nations Development Programme, United Nations Industrial Development Organization, WHO and the Canadian government also provided financial and technical support [26]. Over the last 10 years, an annual budget of US$700,000 has been allocated by the government, and UNICEF has provided a total of about US$2 million since 1998 [41].

## 3. Achievements of the Programme

The surveillance data indicate that China has achieved the global targets for sustainable elimination of IDD: (i) >90% households consuming salt with 15–40 ppm of iodine; (ii) MUIC of the general population within the range of 100–199 μg/L; and (iii) MUIC of pregnant women within the range of 150–249 μg/L [1]. The 2005 National IDD Survey recorded 90.2% of households consuming salt with 20–50 ppm of iodine (the national standard) [42]; by the time of the 2011 survey, this coverage had increased to 95.3% [31]. MUIC of school-age children, taken as a proxy for the general population, exceeded 100 μg/L in both 2005 and 2011: 246 μg/L and 239 μg/L, respectively. Of potential concern is that it exceeded the upper limit of adequate iodine nutrition (100–199 μg/L) as recommended by WHO, UNICEF and ICCIDD in 2007 [1]. Whether this is truly a concern is discussed further below. While the 2005 National Survey did not include an assessment of pregnant women, the 2011 survey did, and the MUIC of pregnant women was found to be in the target range (150–249 μg/L) at 184 μg/L. 

The surveillance data also indicate that many of the targets of the China IDD Elimination Program have been met. By 2010, all provinces and 95% of counties were to have eliminated IDD (Table 2 and Table 3). The 2010 evaluation concluded that IDD had been eliminated in 28 provinces, and in three provinces, it was “almost eliminated”. Ninety seven-point-nine percent of counties were assessed to have met the elimination criteria against the target of 95% [43].

The phenomena of targeting IDD elimination at the county-level data (second level of sub-national stratification after provincial; equivalent to a district) is unique to China. While all countries have adopted the WHO target of national elimination and several collect data on the proportion of households with adequately iodized down to the provincial level or, in one or two cases, down to the district level, none have explicitly set a target of IDD elimination at the county/district level as China has. Moreover, China has undertaken specific evaluations, at the end of each phase of the IDD Elimination Programme, to assess the achievement of these targets.

## 4. Challenges of the Programme

A number of challenges have developed alongside the significant achievements of the Chinese program. 

### 4.1. Achieving Optimal Iodine Nutrition for Both Women and Children through Salt Iodization

China has demonstrated that consumption of adequately iodized salt can eliminate IDD in children. The goiter rate decreased from 20.4% in 1995 to 4% in 2005 as consumption of adequately iodized salt increased from 39%–90.2% in the same period. The Chinese program has also demonstrated how adjustments to the iodine level in salt can be used to bring MUIC levels closer to the optimal range. Reduction of the iodine level in salt from 40–60 ppm to 20–50 ppm in 2000 caused the national MUIC level to fall from 306 μg/L in 1999 to 241 μg/L in 2002. The reduction in the iodized salt standard in 2012 is expected to further reduce MUIC in school-age children, hopefully to within the optimal range recommended by WHO, UNICEF and ICCIDD, of 100–199 μg/L.

China’s quandary, however, is whether it is possible to reduce salt iodine levels sufficiently to bring the MUIC of children into the 100–199 μg/L range while still maintaining the MUIC of pregnant women in their optimal range of 150–249 μg/L. As Table 4 shows, the 2011 National IDD Surveys found only six provinces in which both school-age children and pregnant women were in the optimal range. In the majority of provinces [15], pregnant women were in the optimal range, while school-age children had MUICs between 200 and 299 μg/L, which is classified by WHO, UNICEF and ICCIDD as “above requirements: may pose a slight risk of more than adequate iodine intake in these populations” [1,45].

In 2013, Zimmerman et al. [46] published the results of a study suggesting that the criteria for adequate iodine nutrition in children could be extended from 100–199 μg/L to 100–299 μ/L. This recommendation is based on the finding that there was no change in the prevalence of elevated thyroglobulin or anti-thyroid antibodies comparing children across the ranges of 100–199 μg/L and 200–299 μg/L. The authors conclude that iodine intakes resulting in the “above requirements” (200–299 μg/L) category do not cause thyroid dysfunction in children. Other studies, including in China, also suggest that goiter and thyroid hormone abnormalities seldom occur unless urinary iodine levels exceed 300 μg/L or higher [47,48,49,50,51]. A recently released Chinese study found no evidence of increased thyroid volume in iodine concentrations of 150–249 μg/L and 150–299 μg/L in 7–10- and 11–14-year-old children, respectively [52]. If the range of 100–299 μg/L were adopted by the global community as the optimal range of MUIC for school-age children, both pregnant women and school-age children would have optimal iodine intakes in 20 of China’s 31 provinces, based on the results of the 2011 National IDD Survey (Table 4).

As noted above, current WHO recommendations for the “adequate” MUIC range of pregnant women are 150–249 μg/L [45]. These recommendations were based on a technical consultation in January 2005, the report of which was published in 2007. The recommendation of 150–249 μg/L is based on the FAO/WHO recommended nutrient intake (RNI) for iodine during pregnancy of 250 μg/L and assumptions that 90% of iodine intake is excreted in the urine during pregnancy and that the average volume of urine is 1.5 L per day. The technical consultation also recognized however that “where USI has been effective for at least 2 years, with salt adequately iodized and consumed by more than 90% of the population, it can be reasonably expected that the iodine needs of pregnant and lactating women are covered by their diet, and that the iodine stored in the thyroid glands is sufficient to ensure adequate hormone synthesis and secretion” [53]. This implies, although it is not stated explicitly in the report, that in countries or communities where USI has been effective for at least two years, the MUIC of pregnant women might be lower than the recommended range with no adverse outcomes for women or their children.

The implication for China is that MUIC levels in pregnant women of <150 μg/L may not be of concern in areas where coverage with adequately iodized salt has been >90% for more than two years and the MUIC of school-age children is adequate. Out of the six provinces with the MUIC of pregnant women <150 μg/L in the 2011 National IDD Survey, these criteria are met in all except Shanghai, where coverage with adequately iodized salt was <90% in 2010 [54] and 2011 [31], and Tibet, where iodized salt coverage has never reached 90%.

A study by Ghassabian et al. that investigated the association between low maternal UIC in pregnancy and children’s cognition in a country with optimal iodine status supports this theory. Ghassabian et al. found no association between maternal UIC < 150 μg/L and childhood cognition compared to women with MUIC > 150 μg. The authors hypothesize that this was potentially because many of the pregnant women may have been iodine sufficient prior to conception or during early pregnancy [55]. Similarly, a study in China, also in an iodine-sufficient area, that assessed thyroid function at different levels of iodine intake found no evidence of increased prevalence of thyroid dysfunction (high thyroid stimulating hormone (TSH) or free thyroixine (FT_4_), prevalence of overt and subclinical hypothyroidism and isolated hypothyroxinemia) at 100–149 μg/L compared to 150–249 μg/L, suggesting that iodine intakes below 150 μg/L in early pregnancy had no negative outcomes on the thyroid function of the mother [56].

### 4.2. The Contribution of Processed Foods to Salt and Iodine Intake

WHO, UNICEF and ICCIDD recommend that all edible salt for human and animal consumption, including food processing, be iodized [1,10]. Chinese salt iodization legislation has followed this recommendation. As discussed, China’s salt iodization program has achieved the majority of global and national IDD elimination targets, including MUIC levels at “adequate” or even “above adequate” levels for the majority of pregnant women and children, at both the provincial and national level.

However, a review of the use of iodized salt by the food processing industry by the China National Salt Industry Corporation in 2010 found that only about one-third to a half of salt used in food processing was iodized [57], as this aspect of the legislation is not enforced. Data on the amount of salt used in food processing were obtained from 29 out of 31 provinces, and responses on whether the salt used in food processing was iodized were obtained from 177 out of 190 major food processors in the 29 provinces. Assuming therefore that these data are representative of the national situation, it appears that China has achieved adequate iodine status for the majority of the population despite the fact that the majority of processed foods are not made with iodized salt.

A question has therefore arisen as to whether China could change its legislation to no longer require processed foods to be made with iodized salt, as this component of the legislation is difficult to monitor, hard to enforce and many food processors are opposed to the use of iodized salt. Reasons for opposition to use of iodized salt include concerns about negative organoleptic changes, because the foods are being exported, because the iodine will be lost due to high temperatures in processing, because the salt is used as a food preserver rather than an ingredient and in order to reduce costs [57].

Data from the CNSIC review suggest that the proportion of total edible salt used in food processing is significant. Moreover, the proportion is increasing; while 37% of edible salt was used in food processing in 2009, 50% was used in 2013.

**Figure 1:**
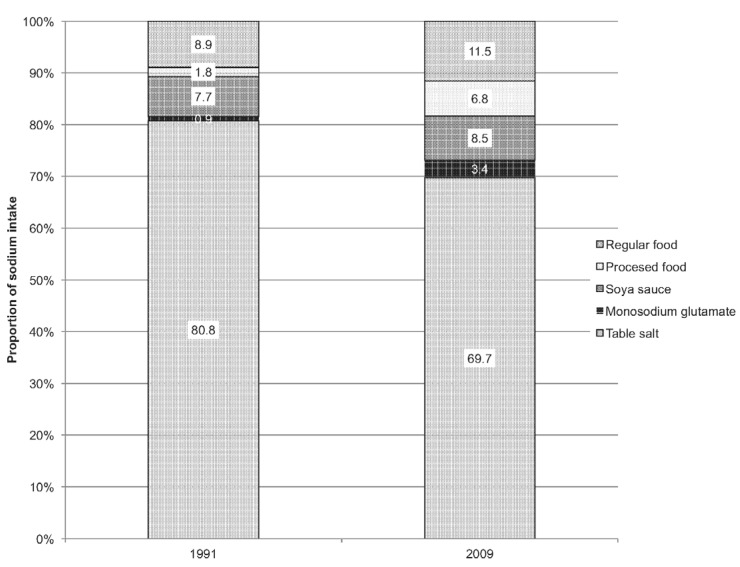
Sources of dietary sodium in China, 1991–2009 [58].

Such increases in the proportion of salt consumed through processed foods are occurring in the context of overall declines in total salt or sodium consumption, including declines in the consumption of direct table salt. Du et al. assessed sodium intake from the China Health and Nutrition Surveys [58]. Sodium intake was found to have decreased from 6.6 g/day in 1991 to 4.7 g/day in 2009. The main source of sodium was salt added during cooking (table salt), but the contribution of table salt to sodium intake decreased from 81% in 1991 to 70% in 2009. Meanwhile the contribution of processed foods and condiments almost doubled from 10.4%–18.7%, as shown in Figure 1. These results indicate that about two thirds of edible salt were consumed as table salt prior to the CNSIC review. Analysis of the data collected from Total Diet Surveys in 2000 and 2009–2011 in 12 of China’s mainland provinces indicated that while weighted salt intake in the home has declined (from 11.8 g/day in 2000 to 9.2 g/day in 2009–2011), sodium intake has only slightly declined (5.6 g in 2009–2011 compared to 6.4 g in 2000) [59]. The trend of all three studies indicates declines in the consumption of table salt and the increasing importance of processed foods as a source of salt.

Rather than abolishing the requirement to use iodized salt for processed food, changing salt consumption patterns may imply a need for the Chinese IDD Elimination Program to pay increasing attention to the iodization of salt used in processed foods, in addition to maintaining high household use of adequately iodized salt.

### 4.3. Addressing the Problem of High Water Iodine Areas

As discussed, the China IDD Elimination Programme has identified 735 townships in 109 counties in 9 provinces as having water iodine levels >150 μg/L. It should be noted however that a comprehensive, national assessment of water iodine levels has not been undertaken. More high water iodine areas may exist that have not yet been identified. Multiple studies have demonstrated negative impacts in high water iodine areas on goiter, MUIC [60,61,62,63,64], children’s intelligence [65] and neonatal thyroid function [66].

Since 2009, non-iodized salt has been supplied to high water iodine areas. The 2014 salt monitoring report indicated that coverage was over 96%. No national surveillance data are currently available on the extent to which this has normalized iodine status however. A study by Lv et al. found that while removal of iodized salt in HWI areas of Hebei province significantly decreased goiter across sex and age groups and decreased MUIC in school-age children from 518 μg/L down to 416 μg/L, it was not sufficient to reduce MUIC to optimal levels [67]. Another study found that in areas with water iodine levels of 150–300 μg/L, 10.5% of the population exceeded the upper limit for iodine intake when iodized salt was consumed, but when non-iodized salt was used, this proportion fell to 1.5%. In areas with water iodine levels ≥300 μg/L, the proportions were 25% and 1.7%, respectively [64]. Investigations of the situation in high water iodine areas therefore concluded: “The supply of non-iodized salt alone cannot ensure adequate iodine nutrition of the residents and water improvement must be adopted, as well” [35,68,69,70].

The 2011/2012 investigation of high water iodine areas [35] concluded that the national cut-off for designation of high water iodine areas should be lowered from 150 μg/L down to 100 μg/L because children had excessive MUIC levels >300 μg/L in all of the study sites when water iodine was 100–149 μg/L. Pregnant women in three out of the four sites also had above requirement MUIC levels >250 μg/L. A revised national standard for high water iodine areas, setting the cut-off point at 100 μg/L, has been developed, but it has not yet been approved. An investigation of water iodine content will be undertaken to identify high water iodine areas based on the new proposed cut-off and estimate how many areas or people may be affected. To date, however, there has been little success in improving water sources in high water iodine areas, partially because the national standard for safe water does not include an iodine indicator [71].

### 4.4. Ensuring High Coverage of Adequately Iodized Salt in All Provinces

From the start of the Chinese IDD Elimination Program, there has been a focus on the achievement of IDD elimination at the sub-national, as well as the national level. A target of the first phase was that 50% of counties would meet criteria to eliminate IDD, and the goal of the third phase was the elimination of IDD in all provinces and 95% of counties. Four provinces, Hainan, Tibet, Qinghai and Xinjiang, have traditionally had low coverage of iodized salt because of extensive availability of raw salt. Sea salt is produced along the coast in Hainan, and extensive rock or lake salt deposits exist in Tibet, Qinghai and Xinjiang. The rock and lake salt in the latter three provinces is of poor quality with high levels of impurities. It is thus illegal to exploit it, even as a source of industrial salt. Local authorities have had difficulty controlling the exploitation of these salt resources, however, with the situation exacerbated by vast distances, sparsely distributed populations and the cost of ensuring supplies of quality iodized salt [72,73,74]. The fourth phase therefore requires only 90% of counties to achieve IDD elimination in these four provinces, whereas in all other provinces, >95% of counties must eliminate IDD.

Achievements of the fourth phase of the China IDD Elimination Programme have not yet been evaluated, but the Salt Monitoring Report of 2014 and the National IDD Survey in 2011 both show that the situation in these four provinces has improved such that coverage of adequately iodized salt is now over 90% in all of these provinces. Moreover, the MUIC of school-age children is adequate (>100 μg/L) in the four provinces [31]. The MUIC of pregnant women is also adequate (>150 μg/L) in all provinces, except Tibet [31]. Improvements in coverage with adequately iodized salt have largely been achieved through a combination of the development of distribution systems for iodized salt by the CNSIC, subsidies for iodized salt from central and provincial governments and education to use iodized salt rather than traditional, local salt. Long-term subsidization will be necessary to maintain the availability and use of adequately iodized salt in those provinces with extensive availability of salt deposits: Tibet, Qinghai and Xinjiang. In Hainan, the government organized the closure of many small salt production facilities and re-developed them for tourism. This removed a significant supply of raw, non-iodized and inadequately iodized salt from the market and boosted coverage of adequately iodized salt from remaining large producers [75]. Few other countries have had similar success in increasing the coverage of iodized salt in salt-producing areas because of the lack of budget or political commitment to adopt the subsidization strategy.

Although the situation in these traditional low coverage provinces has been largely resolved, new low coverage provinces of a completely different nature have emerged. The 2011 IDD Survey recorded household use of adequately iodized salt lower than 90% in two major cities, Beijing and Shanghai, as well as Gansu and Tibet. It was borderline or only just above borderline in Hebei, Qinghai and Tianjin (90%, 90% and 90.1%, respectively). The MUIC of pregnant women was also found to be deficient in Shanghai and Tianjin. Data from the National Salt Monitoring indicate that adequately iodized salt has remained lower than 90% in Shanghai and Tianjin since 2010 and 2012, respectively. In Beijing, coverage increased to 95% in 2014. In the cities of Beijing, Shanghai and Tianjin, the low coverage appears to be due to relaxed availability of non-iodized salt in response to concerns about excess iodine and reports of increased incidence of thyroid disease [76,77,78,79] and the perception that coastal provinces do not need iodized salt because they consume seafood. Various researchers and the NHFPC have attempted to evaluate these concerns. Iodine intakes in four coastal provinces were analyzed from 2007 and 2009 total diet surveys [37]. The National Food Safety Assessment Committee conducted a risk assessment in 2009 [80] and again in 2016 [81]. China participated in a multi-country study to evaluate whether salt iodization can achieve optimal iodine status in all population groups [82] and undertook studies on the safe upper level of iodine intake in adults [83] and optimal urinary iodine ranges in school children [52]. Overall, the data indicate that while the MUIC of school-age children is in the “above adequate” range in several provinces, there is no consistent evidence of negative impacts on thyroid function or increases in thyroid disease that can be attributed to excess iodine intake from salt. Additionally, the NHFPC has attempted to reduce the amount of iodine provided by iodized salt. However, despite evidence illustrating that iodized salt is not causing excess intake or thyroid disease, residents of provinces, particularly in more ‘developed’ provinces, have called for greater access to non-iodized salt. Maintaining the coverage of adequately iodized salt above 90% is likely to be a major challenge of the next phase of the Chinese IDD Elimination Programme.

## 5. Implications for IDD Elimination Program Globally

Some of the challenges experienced by the Chinese IDD Program are not unique to China, and several of the questions being asked in China are applicable to other countries.

### 5.1. Is It Possible for Salt Iodization to Ensure Adequate Iodine Intake in Pregnant Women without Causing Excess in School-Age Children?

This question is of importance because urinary iodine status is often measured in school-age children as the proxy for iodine status in the general population [1]. At the same time, it is recognized that the most important target groups for the elimination of iodine deficiency are pregnant and lactating women, in order to avoid the most significant impacts of deficiency, during pregnancy and amongst newborns and neonates. Pregnant and lactating women are particularly vulnerable because they have significantly higher requirements than school-age children [53]; they are often advised to reduce their salt intake; and newborns have minimal stores of thyroidal iodine and are hence entirely dependent on iodine in breastmilk or infant formula [84].

If it is maintained that even in the contexts of successful USI for some time, pregnant women should have MUIC levels >150 μg/L, data are accumulating that in many contexts, it is not possible for pregnant women to achieve adequate intakes of iodine from iodized salt alone without school-age children receiving excess intakes. Out of six countries in the East Asia and Pacific region that have MUIC data from both school-age children and pregnant women, only in Mongolia are both population groups in the optimal range. In all other countries, children have adequate iodine intake, but pregnant women have insufficient intake (Australia, New Zealand and The Philippines) or children have more than adequate intake and women have adequate intake (Fiji, Indonesia) (Table 5).

In 2011, Wong et al. published an analysis of MUIC data in the WHO Vitamin and Mineral Nutrition Information System as of December 2008 for pregnant women compared to that of school-age children and/or non-pregnant women that was collected from the same location and in the same year. The analysis found that when MUIC of school-age children or non-pregnant women was adequate or above requirements, about 50% of the time, pregnant women had inadequate intake [92]. Regression analysis of the data indicates that in order for predicted MUIC of pregnant women to be between 150 and 250 μg/L, MUIC of school-age children would need to be 178–348 μg/L and MUIC of non-pregnant women would need to be 154–258 μg/L [93]. Both of these ranges would put some groups of school-age and non-pregnant women in the ‘above-requirements’ category.

A multi-country study is currently on-going, under the coordination of Swiss Federal Institute of Technology, the Global Alliance for Improved Nutrition and UNICEF, to determine whether USI can meet the physiological dietary requirements of iodine in women of reproductive age, pregnant women, lactating women and infants up to two years of age without causing excess iodine intake in school children and non-pregnant non-lactating women; preliminary results suggest it can [82]. In addition, WHO is planning to verify or update epidemiological criteria for assessing iodine nutrition in school-age children and pregnant women [94]. The Chinese Institute of Nutrition and Health is undertaking a metabolic balance study, with UNICEF funding, to determine the necessary iodine intake of Chinese pregnant women to sustain optimal maternal and neonatal thyroid function [95]. The Centre for Endemic Disease Control in China Centres for Disease Control is also examining surveillance data from the 2014 IDD Survey to assess the impact of the new iodized salt standards on the MUIC of school-age children and pregnant women [96]. If the various studies confirm that it is not possible to achieve adequate iodine intakes in both population groups, with no risk of excess intake in school-age children, the implication is that salt iodization programs should be adjusted to provide adequate iodine for the general population only, and the higher requirements of pregnant women will need to be met through supplementation or other complementary strategies. Currently, several developed countries have adopted this approach, including Australia [97], New Zealand [98] and the United States [99].

### 5.2. Does Salt Used in Food Processing Need to Be Iodized in Order to Ensure Adequate Iodine Intakes in the General Population?

Although the WHO, UNICEF and ICCIDD recommendation is that all edible salt for human and animal consumption, including salt for food processing, should be iodized, China has achieved IDD elimination in school-age children despite the fact that many processed foods appear not to be made with iodized salt. In contrast, Indonesia [86] and The Philippines [91] have achieved elimination in school-age children despite low coverage of adequately iodized household salt, apparently through iodized salt in processed foods. Numerous other countries have eliminated IDD in school-age children, but data on the extent to which salt for processed food has contributed to this achievement are not available. A further complication exists in Asia where the use of salt-based condiments, such as soya sauce and fish sauce, is common. Such condiments are often consumed in place of “table salt” both in cooking and at the table. Salt is used in the production of these condiments, both as a food ingredient, but also as a preservative. There is widespread resistance within the industry making these condiments use iodized salt because of concerns about changes in organoleptic properties, but also because of cost differences between iodized and non-iodized salt and the large amounts of salt involved [57]. The Thai Ministry of Health currently requires major salt-based condiments (fish sauce, soy sauce and “salt brine”) to be made with iodized salt or to have iodine added directly during the production process [100]. Reportedly, producers of these condiments add the iodine directly; none have elected to use iodized salt because of cost implications [101]. The Thai Ministry of Health is monitoring the situation to see if it is possible to achieve adequate iodine intake through table salt and salt from processed foods only, excluding salt-based condiments. If this appears to be the case, salt-based condiments may be exempted from further iodization requirements in Thailand [102].

Even assuming it is possible to eliminate IDD through table/household salt alone, it is questionable whether this will continue to be the case, as salt intake through processed food increases as countries develop. CNSIC data suggest that 50% of edible salt is used in food processing in China. Data from a selection of other countries indicate similar or even higher proportions of edible salt used for food processing or total sodium consumed from processed foods: Indonesia, 46% [103]; The Philippines, 57% [104]; South Africa, 60% [105]; the United States, 75% [106]; and the United Kingdom, 77% [107]. The potential for the risk of iodine deficiency may be further exacerbated by declines in salt consumption, due to salt reduction strategies for the prevention of non-communicable diseases, such as high blood pressure, heart disease and stroke. Poland adopted mandatory iodization of salt in 1997, and salt monitoring in 1999 indicated that over 90% of households were consuming salt that complied with national standards. Urinary iodine levels in school children have increased from 60.4 μg/L–96.2 μg/L. They do not indicate adequate iodine status, however, possibly because salt for processed foods, which is estimated to make up 40% of salt intake, is not required to be iodized [108].

In order to make decisions about the importance of iodized salt in processed food, countries need improved data on salt intakes, including from processed foods; consumption patterns of processed foods; and the extent to which iodized salt is being used in processed foods.

### 5.3. To What Extent Is Water an Important Source of Iodine and in What Conditions Is Iodized Salt Contraindicated?

China has already demonstrated that water is causing excess iodine intakes for about 2% of the Chinese population. Recent data suggest that even lower levels of water iodine are causing excess iodine intake, so potentially affecting an even larger proportion of the Chinese population. As described, China appears to have been successful at providing non-iodized salt in affected areas, something that other countries may find hard to achieve. Data are not yet available from all affected areas, however, on whether the removal of iodized salt has enabled MUIC levels to return to optimal levels. Data available so far suggest that above a certain level of water iodine, eliminating iodine intake from iodized salt makes little difference, and it is necessary to provide alternative supplies of water in affected areas. It seems likely however that the analysis of China’s surveillance data will enable a range of water iodine cut-off points to be established, within which stopping iodized salt will reduce urinary iodine levels to optimal levels and above which it is necessary to change the water supply.

China’s experience may also provide lessons learned on the best way to identify areas with excess water iodine. If the proposed new “high water iodine” standard is adopted, China will need to undertake an extensive assessment of water iodine content to identify areas meeting the new standard. Evidence already exists that a number of other countries have areas with high water iodine. Sri Lanka has assessed water iodine in its latest IDD survey. While the national median water iodine level was recorded at only 7 μg/L, provincial levels varied from a low of 0 μg/L to a high of 35 μg/L, and individual levels as high as 457 μg/L were recorded [109]. A water iodine content of 578 μg/L was recorded in one district of Indonesia; the extent to which further areas exist with high water iodine is unknown [110]. Finally, a national survey in three zones of Somalia recorded median UICs for non-pregnant women and children of 329 and 416 μg/L, respectively. These excess levels were attributed to household drinking water [111].

Globally, more countries probably need to investigate the existence of high water iodine areas, in particular if MUIC levels do not seem to be explained by household or processed food use of iodized salt. Lessons learned from China could then be used to develop a strategy to achieve optimal iodine status in these areas.

### 5.4. Extrapolating China’s Lessons Learned on Monitoring the Status and Impact of Salt Iodization and IDD Elimination Efforts

A major lesson learned from the China experience is the value and importance of having a comprehensive surveillance system for: (i) sub-national data on household use of iodized salt and MUIC, to ensure equity in high coverage of iodized salt and IDD elimination; (ii) MUIC data from other vulnerable population groups and not only school-age children; and (iii) data on salt, sodium and iodine intakes, including proportions from other sources, e.g., seafood, processed foods and water. Most national salt iodization programs have focused on achieving national targets for household use of iodized salt and urinary iodine levels. Some countries have been able to collect representative data at the provincial or state level, in particular for household use of iodized salt, assessed by qualitative rapid test kits. From the start of its program, however, China emphasized that IDD elimination (including high coverage with iodized and adequately iodized salt, MUIC of school-age children >100 μg/L and goiter rate <5%) should be achieved by every county in China. Provincial and county resources were mobilized for the achievement and assessment of this goal, and today, IDD has been eliminated in 2755 of China’s 2813 counties [31]. This approach has allowed China to quickly identify ”high-risk” and ”low coverage” areas and to address problems specific to these areas.

In 2011, China added the assessment of MUIC in pregnant and lactating women to the surveillance system, enabling the identification of disparities between the iodine status of school-age children and these two high-risk groups. A relatively small number of countries have nationally representative data of the status of pregnant women and even less have sub-nationally representative data. Again, having such data has enabled China to further refine and fine-tune its program. Also in 2011, China collected provincial level data on salt intake and national nutrition, and total diet surveys have collected food consumption data enabling calculations on salt and iodine intake. Further analysis of existing data is needed and planned, which will help inform China’s strategy, particularly in relation to the use of iodized salt in processed foods. Further refinements to the national IDD surveillance system are also under discussion, including, for example, assessment of a thyroid function indicator, such as thyroglobulin, as recommend by Zimmerman et al. [46].

## 6. Conclusions

China’s IDD Elimination Programme demonstrates the effectiveness of mandatory salt iodization in eliminating IDD in school-age children. It also reinforces the importance of achieving equity in high coverage with adequately iodized salt. Several factors have contributed to the success of the Chinese program, including high level political commitment and prioritization, the monopoly structure and government regulation of the Chinese salt industry and the extensive and sophisticate monitoring and evaluation system that has been implemented and refined over the years. 

Chinese experience to date raises questions about whether currently used criteria for indicating optimal iodine status in school-age children and pregnant women are correct and whether MUIC levels <150 μg/L are of concern in pregnant women when they are likely to have entered pregnancy with adequate iodine stores due to successful salt iodization programs. If existing criteria for optimal iodine status are found to be correct, iodine supplementation for vulnerable groups, in addition to universal salt iodization may be required. In addition, data on salt consumption in the Chinese population support global recommendations that salt for food processing, as well as household salt should be iodized in order to ensure adequate intakes. Finally, the Chinese experience highlights the importance of assessing water iodine levels in areas where MUIC levels are higher than can be explained by salt iodization.

## Figures and Tables

**Table 1:** Phases and targets of the National Plan for the Control and Prevention of Key Endemic Diseases [22]. IDD, iodine deficiency disorders.

**Phase 1: 1993–1995**
**Basic access of iodized salt across the country**
(i) 75% national coverage of iodized salt
(ii) 85% coverage of iodized oil capsules among vulnerable groups in iodine-deficient areas
(iii) 50% of counties meet criteria to eliminate IDD
**Phase 2: 1996–2000**
**IDD eliminated at the national level: national coverage of iodized salt increased to 95%**
**Phase 3: 2001–2010**
**IDD eliminated in all provinces and 95% of counties**
**Phase 4: 2010–2015**
**Sustainably eliminate IDD**
(i) above 90% of counties in Hainan, Tibet, Qinghai and Xinjiang eliminate IDD
(ii) 95% of counties in other provinces maintain IDD elimination
(iii) prevention of new cases of cretinism
(iv) maintain iodine nutrition of general population at adequate level

IDD is considered to have been eliminated if four criteria are met: (i) >90% of households consume adequately iodized salt; (ii) >95% of households consume iodized salt; (iii) median urinary iodine concentration (MUIC) of school-age children >100 μg/L; and (iv) goiter rate of school-age children <5%. Criteria for “almost eliminated IDD” and “yet to eliminate” IDD have also been elucidated as shown in Table 2 [23].

**Table 2:** Criteria for elimination of IDD.

Categorization	Criteria
Yet to eliminate IDD	● <90% coverage adequately iodized salt
	OR
	● MUIC of school-age children <100 μg/L
Almost eliminated IDD	● >90% coverage adequately iodized salt
	● >95% coverage iodized salt
	● MUIC of school-age children >100 μg/L
	● Goiter rate of school children <20%
Eliminated IDD	● >90% coverage adequately iodized salt
	● >95% coverage iodized salt
	● MUIC of school-age children >100 μg/L
	● Goiter rate of school children <10%

IDD, iodine deficiency disorders; MUIC, median urinary iodine concentration.

**Table 3:** Achievements of the Chinese IDD Elimination Programme against global and national targets [1,22].

	2005 National IDD Survey [42]	2011 National IDD Survey [31]	2010 End-Line Evaluation [44]
Global Targets for Sustainable IDD Elimination			
>90% household use of salt with iodine content 15–40 ppm	90.2%	95.3%	
MUIC in general population 100–199 μg/L	246 μg/L	239 μg/L	
MUIC in pregnant women 150–249 μg/L		184 μg/L	
China National IDD Elimination Programme Targets			
All provinces eliminated IDD			28 out of 31
95% of counties eliminated IDD			98%

IDD, iodine deficiency disorders; MUIC, median urinary iodine concentration.

**Table 4:** Iodine status of school age children and pregnant women by province, 2011 National IDD Survey [31]. IDD, iodine deficiency disorders.

	Pregnant Women: <150 μg/L	Pregnant Women: 150–249 μg/L	Pregnant Women: ≥250 μg/L	No. of Provinces
School age children: <100 μg/L				0
School age children: 100–199 μg/L	Tibet, Tianjin, Shanghai, Guangdong	Beijing, Xinjiang, Henan, Liaoning, Jilin, Shandong		10
School age children: 200–299 μg/L	Fujian, Guanxi	Hainan, Heilongjiang, Gansu, Hebei, Qinghai, Ningxia, Xinjiang Corps, Zheijiang, Chongqing, Inner Mongolia, Hubei, Hunan, Yunan, Shaanxi, Sichuan	Shanxi	18
School age children: ≥300 μg/L		Guizhou, Jiangxi, Jiangsu, Anhui		4
No of provinces	6	25	1	32

**Table 5:** Median urinary iodine concentration and iodine status of school-age children and pregnant women in selected countries in Asia.

Country (Year)	School-Age Children (μg/L)	Iodine Status [1]	Pregnant Women (μg/L)	Iodine Status [1]
Fiji (2009) [85]	237	Above requirements	227	Adequate
Indonesia (2013) [86]	223	Above requirements	172	Adequate
Mongolia (2011) [87]	171	Adequate	1512	Adequate
Australia (2016) [88]	175	Adequate	116	Insufficient
New Zealand (2011) [89,90]	113	Adequate	85	Insufficient
The Philippines (2013) [91]	168	Adequate	105	Insufficient
